# Melatonin for the treatment of gastroesophageal reflux disease; protocol for a systematic review and meta-analysis

**DOI:** 10.1097/MD.0000000000014241

**Published:** 2019-01-25

**Authors:** Chang Seok Bang, Young Joo Yang, Gwang Ho Baik

**Affiliations:** Department of Internal Medicine, Hallym University College of Medicine, Chuncheon, Korea.

**Keywords:** gastroesophageal reflux, melatonin, reflux esophagitis

## Abstract

**Background:**

Melatonin generated in the gastrointestinal tract has mucosal protective effect with inhibiting gastric acid secretion, while increasing gastrin release, which in turn stimulates the contractility of lower esophageal sphincter. Gastroesophageal reflux disease (GERD) is also known to have association with sleep disturbance. However, melatonin or melatonin receptor agonist has not been included in the treatment of GERD. This study aimed to evaluate the efficacy of melatonin for the treatment of GERD.

**Methods:**

We will search the core databases [MEDLINE (through PubMed), the Cochrane Library, and Embase] from their inception to December 2018 by 2 independent evaluators. The P.I.C.O. is as follows; Patients: who have GERD, Intervention: melatonin or melatonin receptor agonist treatment, Comparison: patients without melatonin or melatonin receptor agonist treatment, Outcome: clinical indices (or crude number or proportion of improvement) for the evaluation of symptomatic improvement which enable comparison of efficacy between patients with melatonin or melatonin receptor agonist and the control group. All types of study design will be sought with full-text will be included. The risk of bias will be assessed using the ROBINS-I tool. Descriptive data synthesis is planned and quantitative synthesis will be used if the included studies are sufficiently homogenous. Publication bias will be assessed with quantitative analyses if more than 10 articles are enrolled.

**Results:**

The results will provide evidence for the efficacy of melatonin or melatonin receptor agonist for the treatment of GERD.

**Conclusion:**

This study will provide evidence of melatonin or melatonin receptor agonist treatment for GERD.

## 1 Introduction

Melatonin, which is mainly generated in the pineal gland has a role for regulation of sleep–wake cycles and circadian rhythms.^[1]^ The synthesis of this hormone shows diurnal/nocturnal fluctuations with low concentrations during the daytime and significant increase during the darkness and seasonal fluctuations with decrease in autumn and winter.^[1–3]^ This hormone has been used to treat sleep disorders or reducing the effect of jet lag promoting the reset of sleep–wake cycles.^[4]^ Seasonally related depression has been also one of the diseases treated with melatonin.^[3]^ In addition to pineal gland, many other organs including gastrointestinal tract, retina, extraorbital lacrimal gland, or bone marrow cells are known to produce melatonin and melatonin receptors also have been discovered in various organs including gastrointestinal tract.^[5–7]^

Melatonin generated in the gastrointestinal tract has mucosal protective effect. It prevents the formation of acute gastric injury and accelerates healing of chronic ulcers with increasing the activity of nitric oxide synthase and cyclooxygenase, which results in the increase of nitric oxide, prostaglandin E2, and mucosal blood flow.^[2]^ It also maintains esophageal mucosal integrity with inhibiting gastric acid secretion, while stimulating duodenal bicarbonate secretion and increasing gastrin release, which in turn stimulates the contractility of lower esophageal sphincter (LES). All these actions have potential for protecting the esophageal mucosa by minimizing contract with acid, bile, or pepsin in animal studies.^[2,8–10]^

Gastroesophageal reflux disease (GERD) is known to have close association with sleep disturbance. Poor quality of sleep is one of the risk factors for GERD symptoms and nocturnal symptom such as night-time heartburn also lowers the quality of sleep and makes it difficult to fall asleep.^[11]^ Treatment of GERD has shown improvement of symptoms not only for night-time heartburns, but also for poor quality of sleep.^[12]^ Although this bidirectional influence between sleep disorders and GERD, the evidence about therapy for targeting of sleep disorders in patients with GERD or both conditions have been scarce. Proton pump inhibitors have been mainly used and recommended for the treatment of GERD; however, melatonin or melatonin receptor agonist has not been included in the treatment of GERD.^[13–16]^ This study aimed to evaluate the efficacy of melatonin or melatonin receptor agonist for the treatment of GERD.

## 2 Methods

This systematic review and meta-analysis will fully adhere to the principles of the Preferred Reporting Items for Systematic reviews and Meta-Analyses (PRISMA-P) checklist.^[17]^ This study was registered at PROSPERO (https://www.crd.york.ac.uk/prospero) on November 2018 (registration number, CRD42018118516) before study was initiated. The approval of institutional review board was exempted due to the characteristics of this study (collecting and synthesizing data from published studies).

### 2.1 Literature searching strategy

MEDLINE (through PubMed), the Cochrane library, and Embase will be searched using common keywords associated with melatonin or melatonin receptor agonist for the treatment of GERD (from inception to December 2018) by 2 independent evaluators (CSB, and YJY). Medical Subject Heading or Emtree keywords will be selected for searching electronic databases. The abstracts of all identified studies will be reviewed to exclude irrelevant publications. Full-text reviews will be performed to determine whether the inclusion criteria are satisfied in the remaining studies, and the bibliographies of relevant articles will be rigorously reviewed to identify additional studies. Disagreements between the evaluators will be resolved by discussion or consultation with a third evaluator (GHB). The detailed searching strategy is described in Table 1.

**Table 1:**
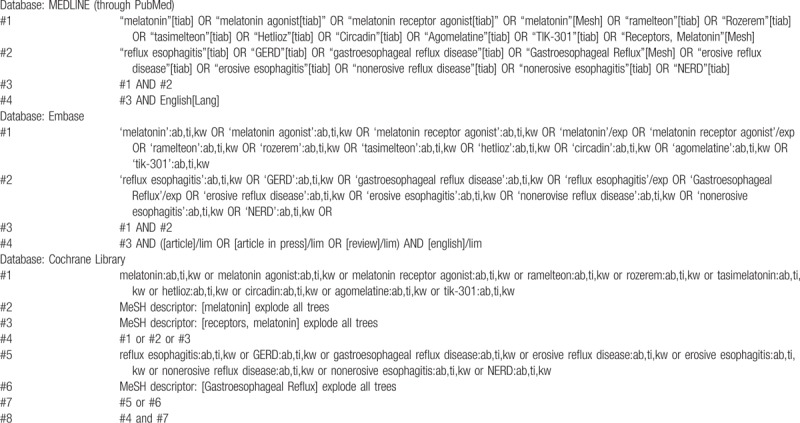
Searching strategy to find the relevant articles.

### 2.2 Selection criteria

We will include studies that met the following criteria: patients: who have GERD; intervention: melatonin or melatonin receptor agonist treatment; comparison: patients without melatonin or melatonin receptor agonist treatment; outcome: clinical indices (or crude number or proportion of improvement) for the evaluation of symptomatic improvement which enable comparison of efficacy between patients with melatonin or melatonin receptor agonist and the control group; study design: all types including randomized, prospective, retrospective studies or case studies; studies of human subjects; and publications in English; full-text publications. Studies that met all of the inclusion criteria will be sought and selected. The exclusion criteria are as follows: review articles; guidelines, consensus documents or expert position papers; comments, letters, brief reports, proceedings, or protocol studies; publications with incomplete data; and meta-analysis articles. Studies meeting at least 1 of the exclusion criteria will be excluded from this analysis.

### 2.3 Methodological quality

The methodological quality of the included publications will be assessed using the Risk Of Bias in Non-randomized Studies-of Interventions (ROBINS-I) tool.^[18]^ The ROBINS-I tool contains 7 domains, including “bias due to confounding” and “bias in selection of participants into the study” at pre-intervention, “bias in classification of intervention” at intervention and “bias due to deviations from intended interventions,” “bias due to missing data,” “bias in measurement outcomes,” and “bias in selection of the reported result” at post-intervention.^[12]^ Each domain is determined to exhibit low-, moderate-, serious-, or critical risk of bias. No information category will be used only when insufficient data are reported to permit a judgement.^[18]^ Overall risk of bias judgement is determined based on the interpretation of each domain level and low risk indicates that the study is comparable to a well-performed randomized trial for all domains being evaluated. Moderate risk of bias indicates the evidence of study is sound for a nonrandomized study but not comparable to a randomized trial (low or moderate risk of bias for all domains). Serious risk of bias indicates the presence of important problems (serious risk of bias in at least one domain, but not at critical risk of bias in any domain). Critical risk of bias indicates the study is problematic to provide any useful evidence (critical risk of bias in at least one domain).^[18]^

Two of the evaluators (CSB and YJY) will independently assess the methodological qualities of all the included studies, and any disagreements between the evaluators will be resolved by discussion or consultation with a third evaluator (GHB).

### 2.4 Data extraction and primary and modifier-based analyses

Two evaluators (CSB and YJY) will independently use the same data fill-in form to collect the primary summary outcome and modifiers in each study, and disagreements between the 2 evaluators will be resolved by discussion or consultation with a third author (GHB).

The primary outcome of this study is the efficacy of melatonin or melatonin receptor agonist for the treatment of GERD. Efficacy can be defined as improvement in the global patient assessment, the visual analog scale of symptoms, indices for the evaluation of symptomatic improvement, such as the Nepean Dyspepsia Index, or any other validated tools indicating overall symptom of patients.^[19]^ The following data will also be extracted from each study, whenever possible; study design, age or ethnicity of enrolled population, sample size, published year, diagnostic method of GERD and assessment tool of outcome (efficacy of melatonin or melatonin receptor agonist treatment).

Narrative (descriptive) synthesis is planned and quantitative synthesis will be used if the included studies are sufficiently homogenous. The common effect size will be extracted from each study, whenever possible and we will also perform sensitivity analyses and meta-regression using the modifiers identified during the systematic review to confirm the robustness of the main result and to identify the reason of heterogeneity.

### 2.5 Statistical analysis

Comprehensive Meta-Analysis Software (version 3, Biostat; Borenstein M, Hedges L, Higgins J and Rothstein H. Englewood, NJ) will be used for this meta-analysis. Heterogeneity will be determined using the *I*^*2*^ test developed by Higgins, which measures the percentage of total variation across studies.^[20]^*I*^*2*^ will be calculated as follows: *I*^*2*^ (%) = 100 × (*Q*−df) /*Q*, where *Q* is Cochrane's heterogeneity statistic, and df signifies the degrees of freedom. Negative values for *I*^*2*^ will be set to zero, and an *I*^*2*^ value over 50% was considered to be of substantial heterogeneity (range: 0%–100%).^[21]^ Pooled-effect sizes with 95% confidence intervals (CIs) will be calculated using the DerSimonian and Laird random effects model meta-analysis and sensitivity analyses will be performed using the Mantel–Haenszel fixed-effect model meta-analysis.^[22]^ These results will be confirmed by the *I*^*2*^ test. Significance will be set at *P = *.05. Publication bias will be evaluated using Begg's funnel plot, Egger's test of the intercept, Duval and Tweedie's trim and fill, and Begg and Mazumdar's rank correlation test.^[23–27]^

## 3 Discussion

This is the protocol of a systematic review and meta-analysis for the efficacy of melatonin or melatonin agonist for the treatment of GERD. The pathogenesis of GERD is complex and sleep deprivation is one of the risk factors of GERD. Sleep disturbance is associated with worsening of symptoms by modulating esophageal perception thresholds for pain in patients with symptomatic erosive esophagitis^[28]^ and nocturnal symptoms of GERD are also known to lower the quality of sleep.^[11]^ Treatment of GERD has shown improvement of symptoms not only for nighttime heartburns, but also for poor-quality of sleep.^[12]^

Although this bidirectional relationship between sleep disorders and GERD, previous studies about sedative for the treatment of GERD have shown conflicting results. Benzodiazepine has shown worsening of symptoms of GERD by reducing the LES pressure, esophageal peristalsis and gastric emptying.^[29–32]^ Alprazolam also led to nocturnal symptoms although no significant effect on LES pressure or esophageal motility but interfering with normal nocturnal acid clearance mechanisms triggered by arousal from sleep due to alprazolam-induced central nervous system depression.^[33,34]^ However, visceral analgesics (pain modulator) such as tricyclic antidepressants, selective serotonin uptake inhibitors, serotonin-norepinephrine reuptake inhibitors or trazodone also have been recommended in patients with refractory GERD who have a physiologic reflux and positive symptom association.^[35]^

In contrast to the sedative agents which suppresses normal acid clearance mechanisms triggered by arousal from sleep, melatonin generated by the enterochromaffin cells in the stomach and intestinal tract (including melatonin agonists) induces sleep through activation of melatonin receptors and has shown esophageal mucosal protective effect by minimizing contract with acid, bile, or pepsin reflux in animal studies.^[2,8–10]^ Improvement of sleep quality by melatonin or melatonin receptor agonists is also expected as one of the mechanisms of alleviating symptoms of GERD by raising perception threshold for pain.^[28]^

This study will provide evidence of melatonin or melatonin receptor agonist for the treatment of GERD.

## Author contributions

**Conceptualization:** Chang Seok Bang.

**Data curation:** Chang Seok Bang, Young Joo Yang, Gwang Ho Baik.

**Formal analysis:** Chang Seok Bang, Young Joo Yang.

**Funding acquisition:** Chang Seok Bang.

**Investigation:** Chang Seok Bang, Young Joo Yang, Gwang Ho Baik.

**Methodology:** Chang Seok Bang.

**Project administration:** Chang Seok Bang.

**Resources:** Chang Seok Bang, Young Joo Yang, Gwang Ho Baik.

**Visualization:** Chang Seok Bang.

**Writing – original draft:** Chang Seok Bang.

**Writing – review & editing:** Chang Seok Bang.

Chang Seok Bang orcid: 0000-0003-4908-5431
